# Development and validation of AI-based automatic measurement of coronal Cobb angles in degenerative scoliosis using sagittal lumbar MRI

**DOI:** 10.1007/s00330-024-10616-8

**Published:** 2024-02-21

**Authors:** Jasper W. van der Graaf, Miranda L. van Hooff, Bram van Ginneken, Merel Huisman, Matthieu Rutten, Dominique Lamers, Nikolas Lessmann, Marinus de Kleuver

**Affiliations:** 1 Diagnostic Image Analysis Group, Radboud University Medical Center Nijmegen, P.O. Box 9101, Nijmegen, 6500 HB The Netherlands; 2 Department of Orthopedics, Radboud University Medical Center Nijmegen, Nijmegen, The Netherlands; 3 Department of Research, Sint Maartenskliniek, Nijmegen, The Netherlands; 4 Department of Medical Imaging, Radboud University Medical Center Nijmegen, Nijmegen, The Netherlands; 5 Department of Radiology, Jeroen Bosch Hospital, ‘s-Hertogenbosch, The Netherlands

**Keywords:** Spine, Scoliosis, Magnetic resonance imaging, Cobb angle, Deep learning

## Abstract

**Abstract:**

**Objectives:**

Severity of degenerative scoliosis (DS) is assessed by measuring the Cobb angle on anteroposterior radiographs. However, MRI images are often available to study the degenerative spine. This retrospective study aims to develop and evaluate the reliability of a novel automatic method that measures coronal Cobb angles on lumbar MRI in DS patients.

**Materials and methods:**

Vertebrae and intervertebral discs were automatically segmented using a 3D AI algorithm, trained on 447 lumbar MRI series. The segmentations were used to calculate all possible angles between the vertebral endplates, with the largest being the Cobb angle. The results were validated with 50 high-resolution sagittal lumbar MRI scans of DS patients, in which three experienced readers measured the Cobb angle. Reliability was determined using the intraclass correlation coefficient (ICC).

**Results:**

The ICCs between the readers ranged from 0.90 (95% CI 0.83–0.94) to 0.93 (95% CI 0.88–0.96). The ICC between the maximum angle found by the algorithm and the average manually measured Cobb angles was 0.83 (95% CI 0.71–0.90). In 9 out of the 50 cases (18%), all readers agreed on both vertebral levels for Cobb angle measurement. When using the algorithm to extract the angles at the vertebral levels chosen by the readers, the ICCs ranged from 0.92 (95% CI 0.87–0.96) to 0.97 (95% CI 0.94–0.98).

**Conclusion:**

The Cobb angle can be accurately measured on MRI using the newly developed algorithm in patients with DS. The readers failed to consistently choose the same vertebral level for Cobb angle measurement, whereas the automatic approach ensures the maximum angle is consistently measured.

**Clinical relevance statement:**

Our AI-based algorithm offers reliable Cobb angle measurement on routine MRI for degenerative scoliosis patients, potentially reducing the reliance on conventional radiographs, ensuring consistent assessments, and therefore improving patient care.

**Key Points:**

• *While often available, MRI images are rarely utilized to determine the severity of degenerative scoliosis.*

• *The presented MRI Cobb angle algorithm is more reliable than humans in patients with degenerative scoliosis.*

• *Radiographic imaging for Cobb angle measurements is mitigated when lumbar MRI images are available.*

**Graphical Abstract:**

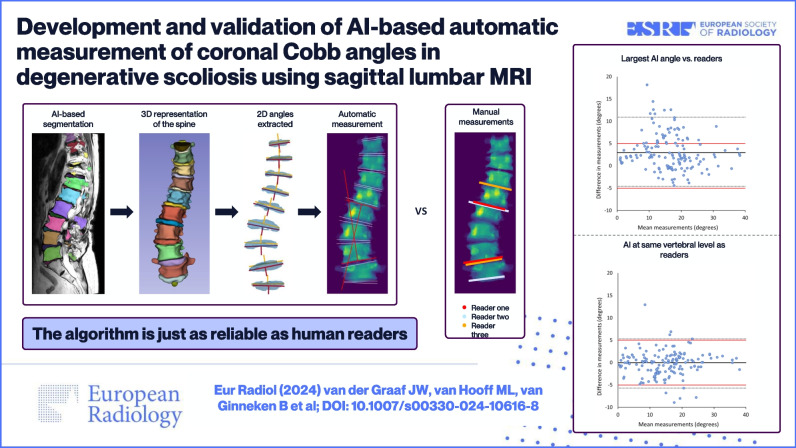

**Supplementary Information:**

The online version contains supplementary material available at 10.1007/s00330-024-10616-8.

## Introduction

Adult spinal deformity (ASD) is a growing problem in an aging society [1, 2]. With the increase in life expectancy and the prevalence of degenerative spinal conditions, the number of individuals affected by ASD has been steadily rising [3]. Degenerative (de novo) scoliosis, a common type of ASD [4], has an estimated prevalence of 36% in the age group of 60 and higher [5], and often causes symptoms such as low back pain or neurogenic leg pain [6]. However, these symptoms can also be assigned to other degenerative spinal diseases [7], potentially leading to misdiagnosis. Accurate assessment and monitoring of degenerative scoliosis progression are crucial for effective treatment planning and patient management.

The Cobb angle [8], a widely accepted metric, is used to evaluate the severity of spinal deformities [9]. Traditionally, the Cobb angle is measured on an anteroposterior (AP) radiograph and assesses the magnitude of spinal curvature as a proxy of disease severity [9]. In clinical settings, patients presenting with low back pain often undergo MR imaging to assess degenerative aspects in the lumbar spine and to evaluate neurogenic structures [10]. The global academic spine community incorporated the use of MRI to assess neural compression in their systematic treatment decision making for patients with degenerative scoliosis [11]. Nevertheless, determining the severity of the curve can pose challenges in MRI, since the 3D volume does not provide one clear overview of the spinal alignment [12].

The development of an automatic method for coronal Cobb angle measurements could increase its reliability [13]. Such algorithms exist for Cobb angle measurements in AP radiographs [14–21], and CT images [22, 23], but do not yet exist for lumbar MRI. The availability of a reliable deep learning algorithm for calculating the Cobb angle from MRI may reduce the necessity to take AP radiographs for patients with degenerative scoliosis when recent MRI scans are available.

In this study, we aimed to develop a novel automatic method designed to measure coronal Cobb angles using sagittal lumbar MRI scans in patients with degenerative scoliosis, and to test its reliability. The proposed automatic measurement approach has the potential to streamline clinical workflows, to reduce subjectivity, and to enhance diagnostic accuracy in the evaluation of degenerative scoliosis.

## Materials and methods

### Dataset

This retrospective study was approved by the institutional review board at Radboud University Medical Center (IRB 2016–2275). Informed consent was exempted, given the use of retrospective anonymized MRI examinations. MRI studies of patients were included if they met all three inclusion criteria:

- MRI made between January 2019 and October 2020.
- “Lumbar musculoskeletal screening” or “Lumbar neuro screening” as MRI study description. These MRI study protocols are used for patients with low back pain or neurogenic pain and reflect a type of exam for which readers could benefit from automatic Cobb angle measurement.
- MRI study contained at least one MRI series made with high resolution sagittal T2 SPACE (Sampling Perfection with Application-optimized Contrasts using different flip angle Evolution) sequence.
- The words “scoliosis” or “spinal deformity” were mentioned in the radiology report.

The inclusion high-resolution (voxel size 0.90 × 0.47 × 0.47 mm) T2 SPACE sequence images was done to enable adequate visualization not only in the sagittal plane but also in the coronal plane. This ensured that manual measurement could be done on the MRI images in the coronal plane. All MRI studies also contained a standard resolution (voxel size 3.29 × 0.59 × 0.59 mm) sagittal lumbar T2 scan. All images were obtained on a 1.5-T MRI system (Avanto, Siemens Healthineers Nederland B.V.).

### Spine segmentation algorithm

**Fig. 1:**
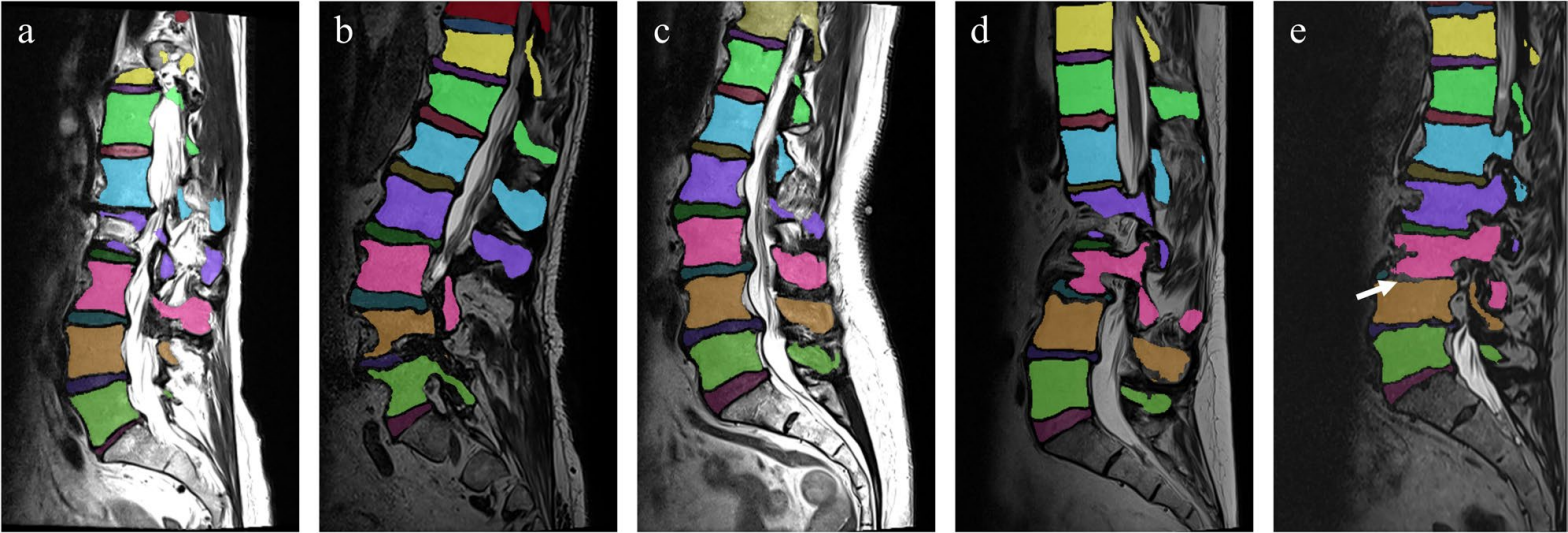
Five examples of sagittal MRI images with their corresponding automatically generated segmentation masks. The different colors represent the different anatomical labels predicted by the segmentation algorithm. Example **e** shows the image of the only IVD in this study with a substantial segmentation error that possibly affected the Cobb angle measurement. The segmentation error is highlighted with a white arrow. The MRI scans of examples **a** to **e** correspond to the examples shown in Figs. 3 and 5

All vertebrae and intervertebral discs (IVDs) within the MRI scan volume were automatically segmented using a 3D AI algorithm, including possible transitional vertebrae (e.g., L6) [24]. Each vertebra and IVD were segmented separately with a unique anatomical label. The algorithm employed a patch-based iterative scheme to segment one vertebra and the corresponding inferior IVD. The architecture of the algorithm is based on the popular 3D U-net structure, which was developed specifically for 3D image segmentation tasks [25]. Examples of the results of this segmentation algorithm are shown in Fig. 1. All segmentations were evaluated on essential errors that could possibly affect the Cobb angle measurements by a medical imaging expert (J.G., 4 years of experience). Essential errors included missing IVDs, too many IVDs, or when the general shape of the IVD is incorrect.

The algorithm was trained on the publicly available SPIDER dataset, a multicenter dataset containing 447 MRI series from 218 patients suffering from low back pain [24]. None of the patients included for clinical validation of the Cobb angle algorithm were present in the SPIDER dataset.

### Cobb angle algorithm

When measured manually on conventional AP views of the spine, the Cobb angle is determined by drawing lines parallel to the upper and lower endplates of the most tilted vertebrae and measuring the angle between these lines [8]. However, in this study, a different approach was taken. Instead of using the upper and lower endplates, the IVDs were utilized to determine the Cobb angle in 3D. This choice was motivated by the fact that IVDs have a simpler geometric shape compared to the vertebrae, making the algorithm simpler and therefore more robust. It was hypothesized that using the upper and lower halves of IVDs would yield similar measurements since they are parallel to the vertebral endplates.

All individual IVD masks were extracted from the spine segmentation results. The masks were converted into a surface mesh, consisting of faces and vertices, using a marching cube algorithm [26]. Principal component analysis (PCA) [27] was used to fit a 3D plane through the IVD. Each plane is comprised of a normal vector and center-of-mass of the IVD. To compose the normal vector, PCA was applied on the vertices of the IVD. This resulted in three eigenvalues and eigenvectors describing the three principal components of the IVD. The eigenvector with the smallest eigenvalue corresponded to the normal vector of the IVD. The center-of-mass was computed by averaging all vertices. This plane was not necessarily parallel to both vertebral endplates; therefore, it was used to split the vertices of IVD surface in an upper and lower half. In similar fashion, 3D planes were fitted through the upper and lower half of the IVD. Splitting the discs and defining planes through the upper and lower half separately ensures that the planes are parallel to the vertebral endplates. These two 3D planes were projected onto the coronal plane after which the angle of the plane in relation to the image-coordinate system could be extracted.

**Fig. 2:**
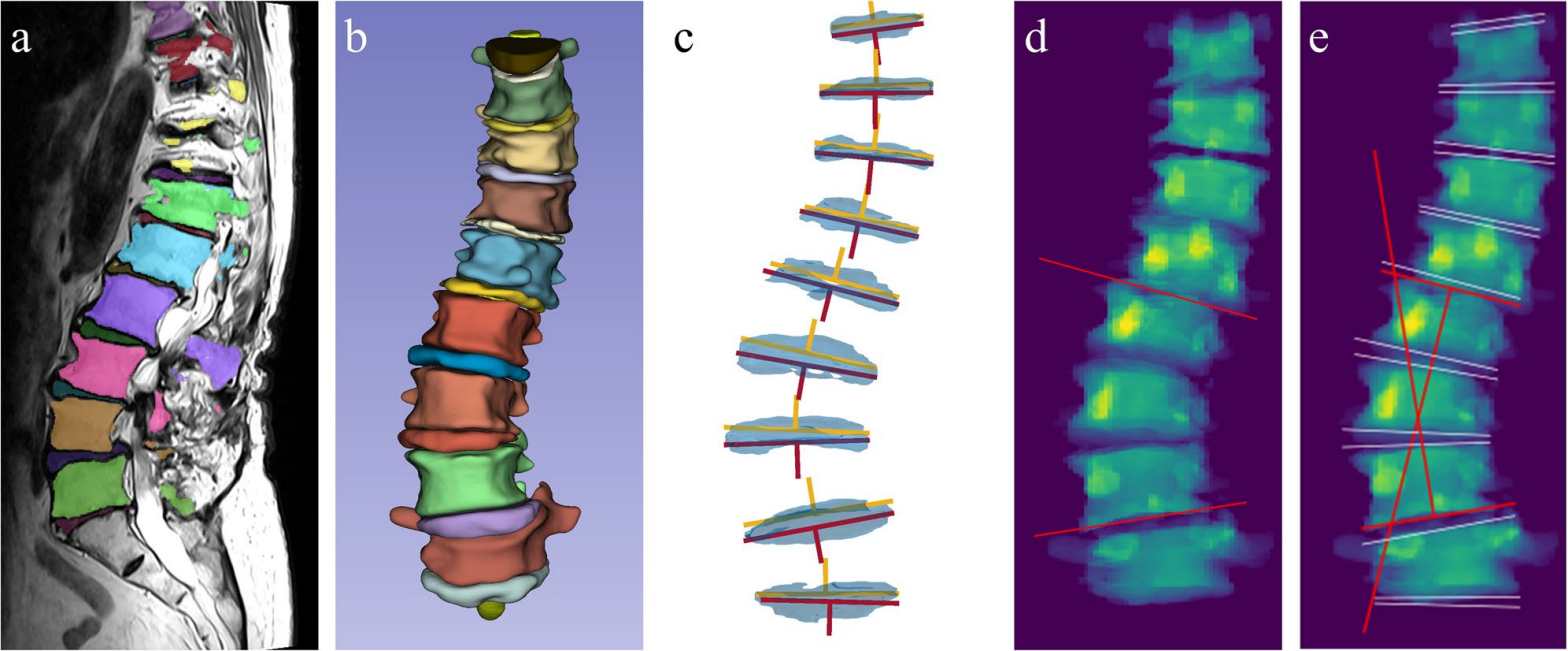
Visualization of the different steps of the Cobb angle algorithm. **a** Automatically generated segmentation visualized on the sagittal MR image of the lumbar spine. **b** Same segmentation visualized in 3D in the coronal plane. **c** Coronal view of all intervertebral discs with the upper (yellow) and lower (red) planes shown as a 2D line and the normal vector. **d** Example of a manual measurement on the 2D coronal projection image. **e** Visualization of the automatic measurement on the 2D coronal projection image

The upper and lower angle of each individual IVD was computed, which corresponds with the angle of each lower and upper vertebral endplate respectively. The algorithm extracts all possible Cobb angles by calculating the difference between the angle of the upper vertebral endplates as top level and the angle of the lower vertebral endplates as bottom level. Since the Cobb angle is defined by the two most tilted vertebrae, the largest difference between two measurements is considered to be the Cobb angle. An overview of the different steps of the algorithm is shown in Fig. 2.

### Clinical reliability

**Fig. 3:**
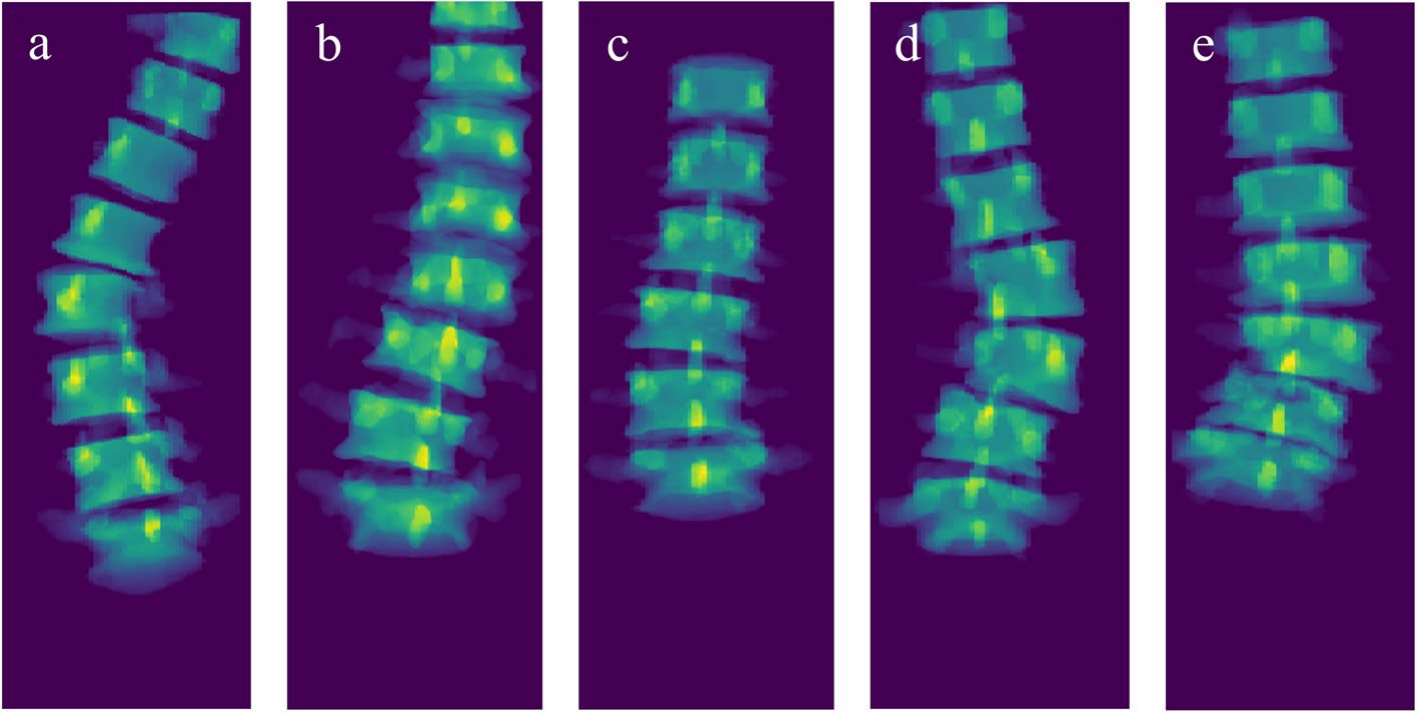
Five examples of the 2D coronal projection images of the flattened segmentation mask. The MRI scans of examples **a** to **e** correspond with the examples shown in Figs. 1 and 5

To assess the reliability of the automated Cobb angle algorithm, the results were compared with manual measurements performed by three experienced readers. The readers consisted of two musculoskeletal radiologists (M.R. and M.H.) and one spine surgeon (D.L.) with 26, 7, and 6 years of experience respectively. The manual measurements were performed on 2D coronal projection images derived from the spine segmentation. These images were generated by projecting the 3D masks of the vertebrae onto the coronal plane which mimics an AP radiograph. Examples of these images are shown in Fig. 3. For their measurements, the readers only needed to draw two lines parallel to the endplates of the two most tilted vertebrae. The Cobb angle was computed by calculating the angle between the two drawn lines. The manual measurements were collected using a reader study which was hosted on the Grand-Challenge.org platform.

For the comparison analysis, both the top and bottom vertebral levels chosen by the readers were considered, along with their corresponding angles. The algorithm, by default, provided the largest possible Cobb angle, which aligned with the traditional measuring method. However, the algorithm also extracted the Cobb angle at all the specified levels, mirroring the approach taken by the expert readers. This enabled a threefold comparison: first, evaluating whether the correct vertebral levels were chosen; second, determining whether the algorithm accurately calculated the Cobb angle at the levels selected by the readers; and third, comparing the Cobb angle automatically measured at the consensus level to the average reader measurement. The consensus level is defined as the median top and bottom vertebral level chosen by the readers. For example, if two readers chose L3 as the bottom vertebral level and one reader chose L4, the bottom consensus level would be L4. If all three readers choose a different level, the consensus level would be the middle level of the three chosen levels.

The combined performance of the algorithm and human readers was simulated by computing the difference between the manual and automatic measurements. If this value was less than 5°, which is considered a clinically acceptable measurement error for the Cobb angle [9], we regarded it as a successful automatic measurement that the reader would have concurred with. If the measurement difference was more than 5°, we regarded it as a measurement that the reader would have rejected and would have manually repeated. The data used for this comparison are the automatic measurements at the same vertebral level of the manual measurements.

The MRI series used for clinical validation were all obtained with a high-resolution sagittal SPACE MRI sequence, which is a single slab three-dimensional turbo spin echo (TSE) sequence with a slab selective, variable excitation pulse. These scans ensured that the coronal 2D projection images for manual annotation were of high enough resolution to make an accurate measurement. However, this is not standard practice in most hospitals. All MRI studies also included standard resolution (voxel size 3.29 × 0.59 × 0.59 mm) T2 sagittal lumbar MR scans. The algorithm also measured the largest possible Cobb angle on these standard resolution scans to assess whether it would yield similar results on more commonly available resolution images compared to the high-resolution SPACE sequence images.

### Statistics

Patient demographics (age and gender) were derived from the DICOM-headers. Continuous variables (age, Cobb angles) were described using means and standard deviations (SD) and categorical variables (gender) with counts and percentages. The assessment of agreement between all measurements was performed by calculating the mean absolute error (MAE) between measurements and by using the intraclass correlation coefficient (ICC) and the 95% confidence interval (CI). The ICC is a metric ranging from 0 to 1, where values below 0.50 indicate poor reliability, values between 0.50 and 0.75 suggest moderate reliability, values between 0.75 and 0.90 indicate good reliability, and any value above 0.90 signifies excellent reliability [28]. The results were visualized using Bland–Altman plots with the corresponding upper and lower limits of agreement. These limits of agreement can be compared to the clinically accepted measurement error of the coronal Cobb angle, which is 5° [9]. Statistical analysis was done using JASP (Version 0.17.3) [29].

### Code and data availability

The trained segmentation algorithm (https://grand-challenge.org/algorithms/spider-baseline-iis/), the complete segmentation code-base (https://github.com/DIAGNijmegen/SPIDER-Baseline-IIS), and all segmentation training and validation data (https://zenodo.org/records/10159290) are publicly available [24]. The Cobb angle algorithm is available on https://github.com/DIAGNijmegen/cobb-angle-algorithm.

## Results

**Fig. 4:**
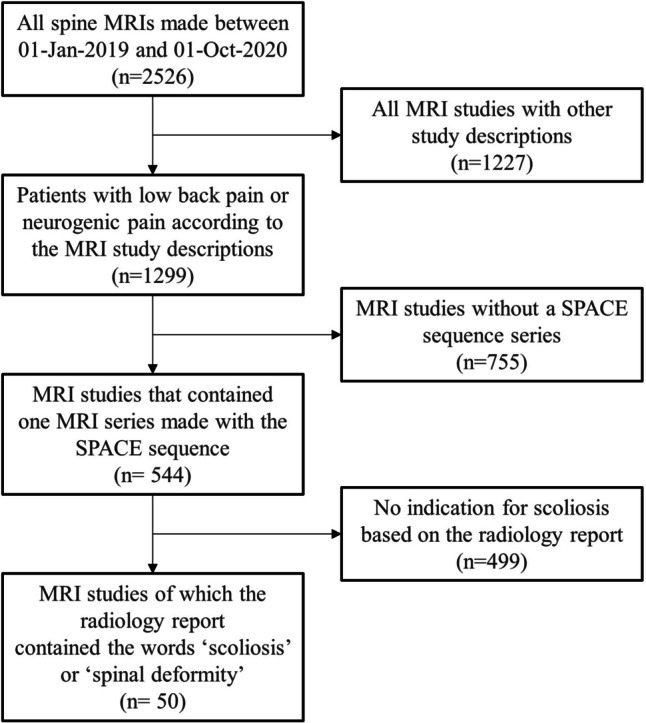
Inclusion flowchart of the MRI studies used for clinical validation

**Fig. 5:**
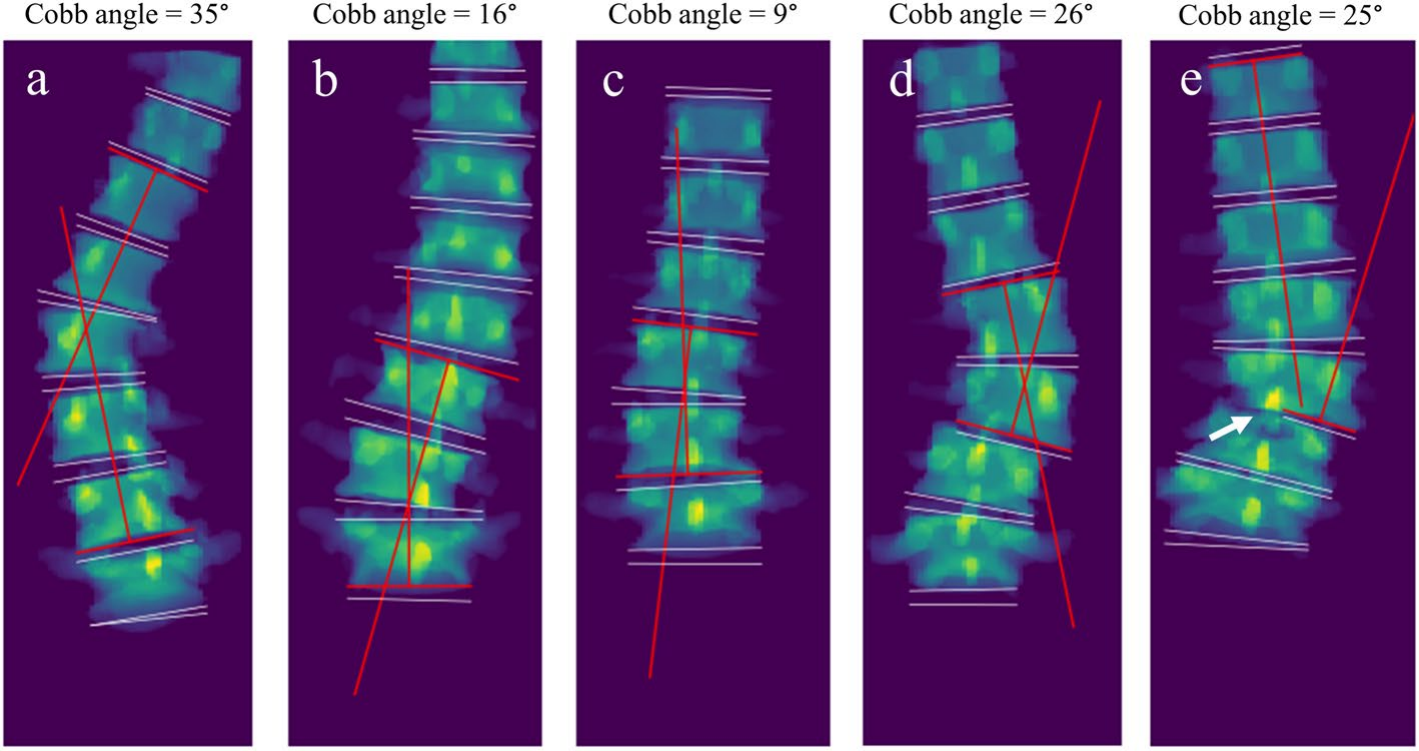
Five examples of the largest automatic Cobb angle measurements depicted on the 2D projection images of the flattened segmentation mask. The white lines represent the planes fitted through the upper and lower half of each intervertebral disc. The red lines are the planes between which the largest Cobb angle is measured. Example **e** shows the image of the only IVD in this study with a substantial segmentation error that possibly affected the Cobb angle measurement. The segmentation error is highlighted with a white arrow. The MRI scans of examples **a** to **e** correspond with the examples shown in Figs. 1 and 3

A total of 50 MRI studies of 50 unique patients that met all inclusion criteria was used for analysis (Fig. 4). The mean age of the patients was 60 (SD 15.6) years, with 35 (70%) females. In one out of the 50 cases, which included 401 IVDs, an error was made in the segmentation of one IVD which possibly affect the Cobb angle measurement. This case is illustrated in Figs. 1e, 3e, and 5e. Two patients had metal pedicle screw and rod implants. In both cases, no significant segmentation errors were observed, resulting in a normal Cobb angle measurement. These two cases are displayed in Supplementary Material A.

**Fig. 6:**
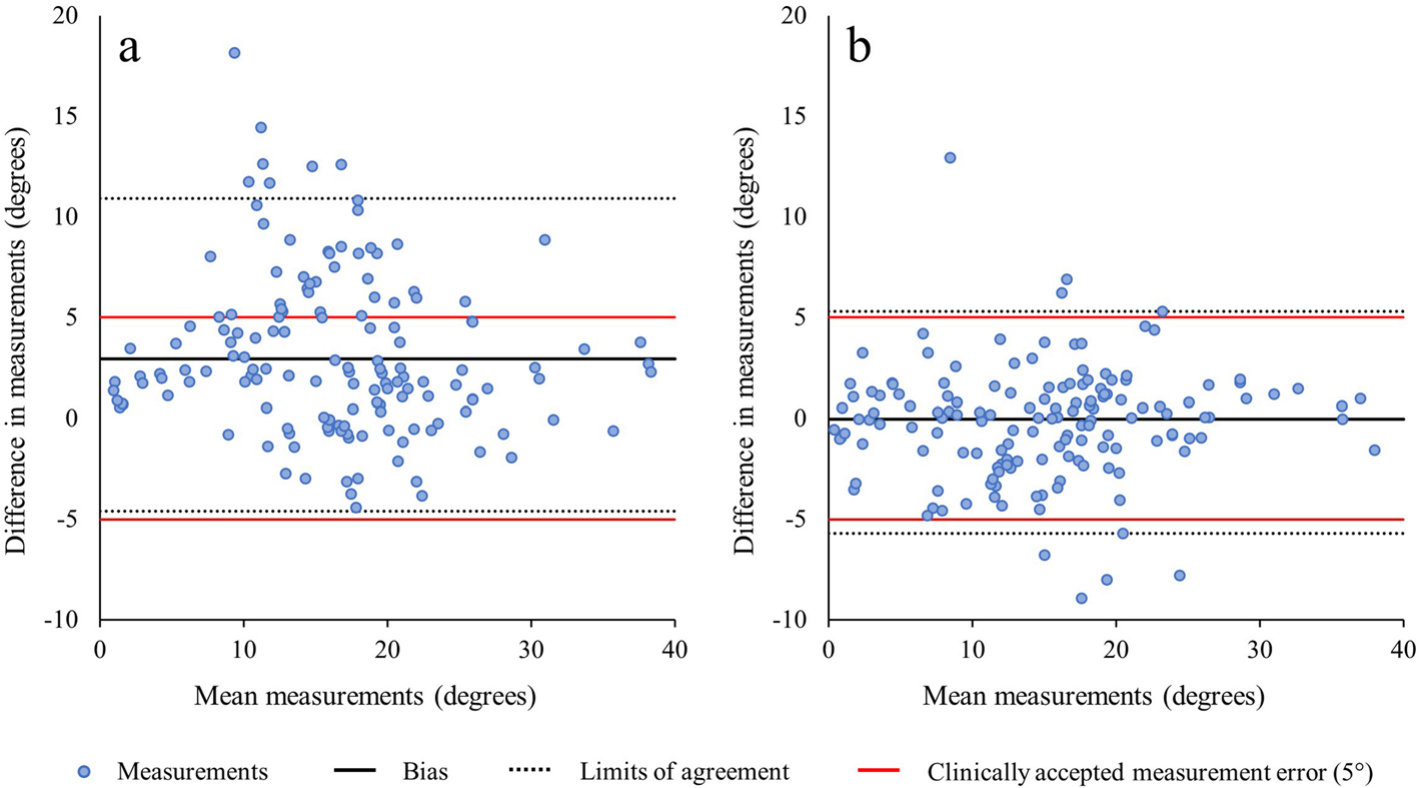
Bland–Altman plots of the manual measurements of the three readers compared to the largest automatic measurement (**a**), and at the exact same chosen vertebral levels as the manual measurements (**b**). The red lines represent the clinically accepted measurement error of 5°. The black line represents the calculated measurement bias and the dotted lines represent the corresponding upper and lower limits of agreement

The mean manually measured Cobb angle across all three readers was 14.8° (SD 8.2°). Supplementary Material B shows a figure which displays the distribution of the average manually measured Cobb angles. The MAE between manual measurements of all three readers was 2.7° (SD 1.7°) with ICCs ranging from 0.90 (95% CI 0.83–0.94) to 0.93 (95% CI 0.88–0.96). When taking the maximum angle per scan, the automatic measurements found a mean Cobb angle of 18.0° (SD 7.7°). The MAE and the ICC, compared to the average manually measured Cobb angles, were 3.6° (SD 3.1°) and 0.83 (95% CI 0.71–0.90). Five examples of the automatic measurements are shown in Fig. 5. The ICCs calculated between the different measurements are shown in Table 1. The largest Cobb angle found by the algorithm compared to all manual measurements are visualized in a Bland–Altman plot (Fig. 6a), with a bias of + 3.2° and with upper and lower limits of agreement of 10.9° and − 4.6°, respectively.

**Table 1:** Overview of the intraclass correlation coefficients (ICCs) and the corresponding 95% confidence interval (CI)

ICC (95% CI)	Automatic	Automatic consensus	Reader 1	Reader 2	Reader 3	Mean manual
Automatic	1					
Automatic consensus	0.88 (0.80–0.93)	1				
Reader 1	0.80 (0.68–0.88)	0.93 (0.87–0.96)	1			
Reader 2	0.84 (0.74–0.91)	0.89 (0.82–0.94)	0.90 (0.83–0.94)	1		
Reader 3	0.76 (0.62–0.86)	0.91 (0.85–0.95)	0.93 (0.88–0.96)	0.90 (0.83–0.94)	1	
Mean manual	0.83 (0.71–0.90)	0.94 (0.89–0.97)	0.97 (0.95–0.98)	0.961 (0.93–0.98)	0.97 (0.95–0.98)	1

In measuring the Cobb angle, the three readers chose the same upper vertebral level in 42% (21/50) of the cases. The agreement on the lower vertebral level was 32% (16/50) and in 18% (9/50) of the cases all three readers performed the exact same measurements with the same top and bottom vertebral level. Table 2 shows the results of the chosen vertebral level by the three readers compared to the selected vertebral levels by the algorithm (i.e., largest Cobb angle). When the algorithm measured the Cobb angles at consensus positions, defined as the median vertebral levels chosen by the readers, the mean Cobb angle was of 15.5° (SD 8.1°). Compared to the average reader measurement, there was a MEA of 2.1° (SD 1.9°), and an intraclass correlation coefficient of 0.94 (95% CI 0.89–0.97).

**Table 2:** Differences in chosen vertebral levels between readers and the largest Cobb angle found by the algorithm expressed in a percentage (number of cases out of 50). The cases are categorized based on the absolute difference between the reader and the algorithm, combined for both the top and bottom vertebral level

Vertebral level agreement (%)	Reader 1	Reader 2	Reader 3
Exact same level	18% (*n* = 9)	20% (*n* = 10)	34% (*n* = 17)
One level difference	36% (*n* = 18)	38% (*n* = 19)	32% (*n* = 16)
Two levels difference	28% (*n* = 14)	20% (*n* = 10)	26% (*n* = 13)
More than two levels difference	18% (*n* = 9)	22% (*n* = 11)	8% (*n* = 4)

The algorithm was also used to extract the angles at the exact chosen vertebral levels of the three readers. The ICCs of the algorithm and the readers were 0.93 (95% CI 0.88–0.96) for reader 1, 0.97 (95% CI 0.94–0.98) for reader 2, and 0.92 (95% CI 0.87–0.96) for reader 3. The MAE was 2.0° (SD 1.3°). Of the 150 manual measurements, 50 per reader, eight (5.3%) had an absolute measurement difference, which exceeded the clinically acceptable measurement error of 5°. Images of all eight cases can be found in Supplementary Material C. All manual measurements compared to the automatic measurement at the exact same chosen vertebral levels are visualized in a Bland–Altman plot (Fig. 6b), with a bias of − 0.2° and with upper and lower limits of agreement of 5.3° and − 5.7° respectively. Lastly, the Cobb angles found in the low-resolution T2 MR scans compared to the high-resolution MR scans showed an ICC of 0.95 (95% CI 0.91–0.97).

## Discussion

This study presents a newly developed AI-based algorithm that reliably computes the coronal Cobb angle on lumbar MRI scans for patients with degenerative scoliosis, when compared to three expert readers. When the algorithm extracts the largest possible Cobb angle, good agreement (ICC between 0.76 and 0.84) and a measurement bias of + 3.2 was achieved compared to manual measurements. However, in 82% of the cases, the readers disagreed on the vertebral level at which the measurements should be done. When the angle is measured at the same vertebral levels as chosen by the expert readers, there is excellent agreement (ICCs between 0.92 and 0.97), slightly higher even than the agreements between readers (ICCs between 0.90 and 0.93). The mean absolute error (MAE) of these measurements was 2.0° (SD 1.3°), which is slightly lower than the MAE between readers (2.7°, SD 1.7°). However, both are well below the clinically accepted measurement error of 5° [9]. These results not only confirm the consistency of our automated approach with manual measurements but also positions it as a reliable tool within the established bounds of clinical significance. Our findings suggest that our automated method holds promise for clinical application, offering a level of precision comparable to the inherent variability observed among human readers.

Several algorithms have been described in literature which automatically measures Cobb angles on conventional radiographs [14–19]. Liu et al presented a similar approach where AI-based segmentation algorithms are used to provide a mask of the vertebrae radiographic images, which is used for rule-based calculations to determine the Cobb angle [15]. They report a MAE of < 3°. Other studies that used different deep learning approaches compared to the presented algorithm report MAE scores ranging from 1.2° to 3.3° [15, 17–21], which are similar to the results in this study. These measurement errors are not dissimilar to intra- and interobserver variability of manual measurements reported in literature which range from 1.4° to 5.1° [9]. Note that these studies all use conventional radiographs in patients with adolescent idiopathic scoliosis, making it difficult to compare results. This study is the first to present an AI-based algorithm for measuring Cobb angles in degenerative scoliosis based on MRI.

The advantage of using MRI to determine the Cobb angle is that it is measured using a 3D representation of the spine which is projected on the 2D coronal plane, similar to the research of Huo et al [22]. It may be difficult to determine the Cobb angle of lordotic vertebrae (e.g., L4 or L5) since the vertebral endplate is not clearly delineated due to its tilt. Our method finds a 3D angle for each vertebral endplate of which the coronal angle can easily be extracted, which is not influenced by any sagittal tilt. Moreover, the sagittal Cobb angle could also be simultaneously calculated using this method. Lastly, this method could be extended to calculate axial rotation of each vertebra as well, providing a full geometric overview of a complex scoliotic spine. Additional research is required to determine whether the presented method is useful for other spinal alignment measurements.

This study has limitations. First, we did not compare the algorithm to manual measurements on conventional radiographs. This comparison was not performed for two reasons. First, the field of view of radiographs is generally larger than that of a MRI scan. This could potentially lead to a different measurement when the curve continues outside the MRI’s field of view, since no full spine MRI scans were available. Second, the MRI scans were made with the patient laying in supine position whereas conventional radiograph are frequently made in standing position. It is known that Cobb angles measured on images in standing position are larger compared to the supine position, which could make accurate comparison difficult [30]. There are potential ways to translate a Cobb angle measured made in supine position to a standing position [30]. These methods were not tested since the focus of this research was to develop and test a new Cobb angle algorithm for MRI. Moreover, this presented algorithm could still be utilized to monitor curve progression over time.

A second limitation is related to the use of intervertebral discs (IVDs) to compute the Cobb angle. The accuracy of the algorithm depends on the accuracy of the IVD segmentations, which might be negatively affected by abnormalities such as irregular IVDs or severely degenerated IVDs. Manual review of all IVD segmentations revealed that only one of 401 was wrongly segmented, resulting in a possibly incorrect Cobb angle measurement (Fig. 5e). This IVD was completely collapsed on one side, resulting in an IVD segmentation that only covered half of the vertebral endplate. No significant errors were present in any of the other IVD segmentations. Subsequently, the algorithm works under the assumption that a plane fitted through the upper and lower half of the IVD its surface would be parallel to the vertebral endplate. However, this might not be the case in wedge-shaped IVDs or other irregularities [31], possibly causing inaccurate measurements. Since the surface of the annulus is not uniform along the whole wedge-shaped IVD, the fitted planes could be tilted towards the highest side of the IVD, resulting in the plane not being parallel to the endplate. We chose not to alter our algorithm to avoid unnecessary complexity, since this would make it more susceptible to errors in cases of pathological IVDs (e.g., Schmorl nodes or herniations). The observed excellent agreements between measurements, over a wide range of different Cobb angles, suggest that any potential impact on accuracy is likely minimal.

A third limitation is related to the type of deformity for which the AI-based algorithm has been developed. This research is not generalizable to types of scoliosis other than degenerative scoliosis, since only lumbar MRI scans were used in this study [32]. In, for example, adolescent idiopathic scoliosis, a full spine view is essential for determining the curve severity [33]. Nevertheless, the excellent agreement between the automatic and manual measurement proves the reliability of the presented method. Consequently, it is expected that when the segmentation algorithm is trained and validated on full spine MRI data, our method will likely yield similar results. This assumption should of course be confirmed with additional research.

We conclude that the Cobb angle can reliably be measured by using the newly developed automatic AI-based algorithm on routine MRI in patients with degenerative scoliosis. This study also showed that experienced readers often disagree on which vertebral levels the Cobb angle should be measured. The presented algorithm ensures that the maximum Cobb angle is consistently measured. We therefore postulate that the AI algorithm is more reliable than the manual Cobb angle measurements on MRI in patients with degenerative scoliosis, possibly mitigating the need of conventional radiographs for determining curve severity.

### Supplementary Information

Below is the link to the electronic supplementary material.

Supplementary file1 (PDF 414 KB)

## Abbreviations

AP: Anteroposterior
ASD: Adult spinal deformity
CI: Confidence interval
DS: Degenerative scoliosis
ICC: Intraclass correlation coefficient
IVD: Intervertebral discs
MAE: Mean absolute error
PCA: Principal component analysis
SD: Standard deviations
TSE: Turbo spin echo

## Declarations

### Guarantor

The scientific guarantor of this publication is Marinus de Kleuver.

### Statistics and biometry

One of the authors has significant statistical expertise.

### Methodology

• retrospective

• experimental

• performed at one institution
