# Supplementary material for: Development and validation of AI-based automatic measurement of coronal Cobb angles in degenerative scoliosis using sagittal lumbar MRI
Source: Eur Radiol. 2024 Feb 21;34(9):5748–57. doi: 10.1007/s00330-024-10616-8 (PMC11364572; doi:10.1007/s00330-024-10616-8)
Supplement: Supplementary file 1 — Supplementary file1 (PDF 414 KB) [file 330_2024_10616_MOESM1_ESM.pdf]

Development and validation of AI-based Automatic Measurement of Coronal Cobb Angles in Degenerative Scoliosis Using Sagittal Lumbar MRI

<u>Electronic Supplementary Material (ESM)</u>

Eur Radiol (2024) van der Graaf JW, van Hooff ML, van Ginneken B et al.

Supplementary material A:

Both a mid-sagittal MRI slice with it's corresponding segmentation mask, and the automatically generated Cobb angle measurements of the two included cases where metal hardware is present.

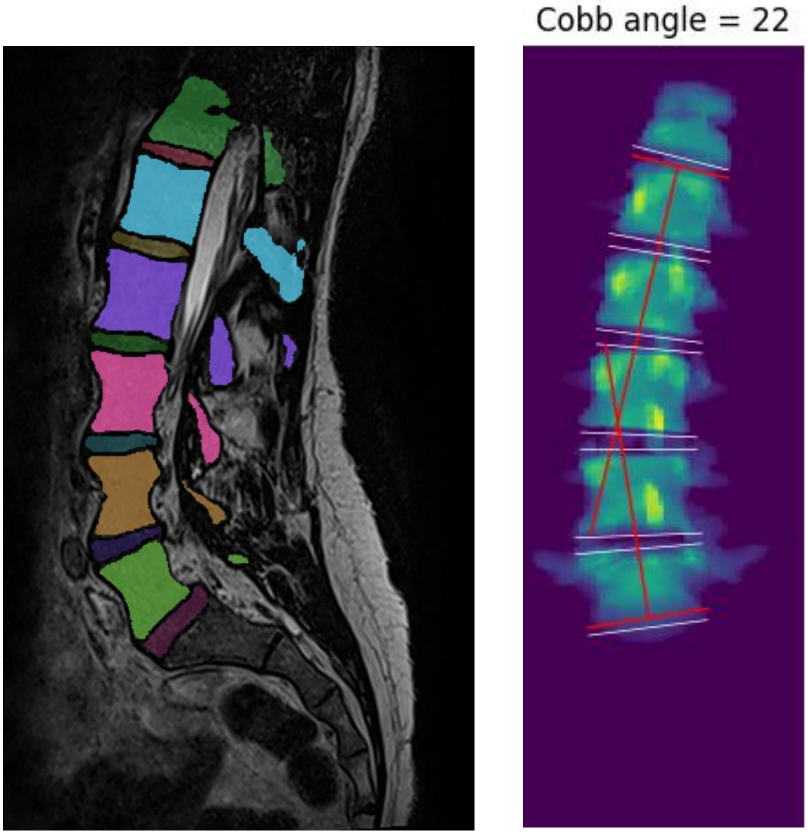

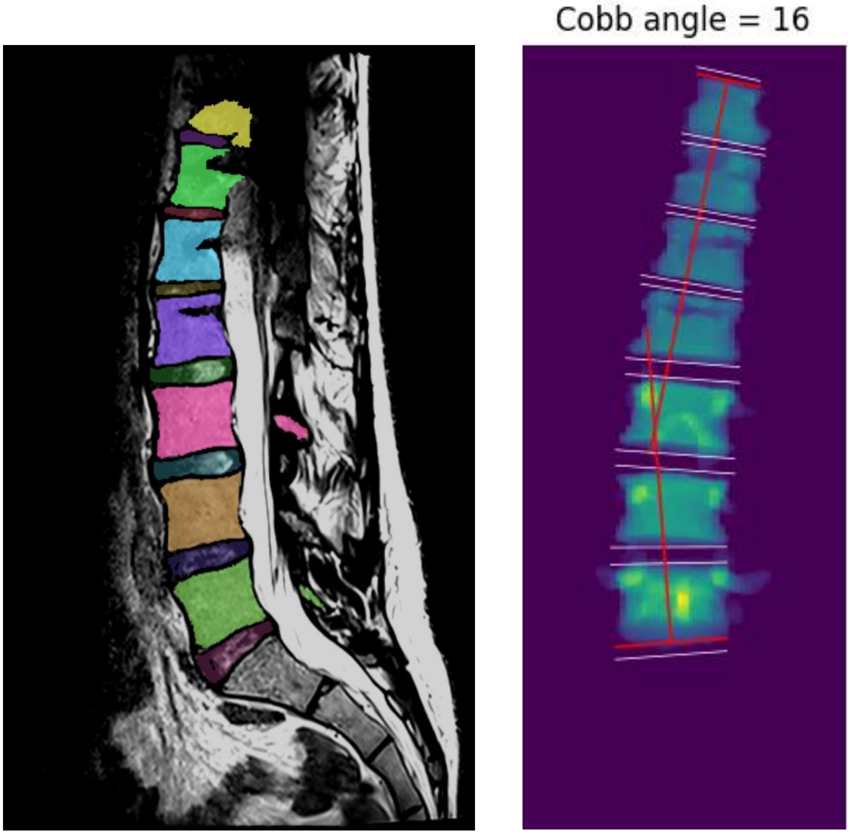

Eur Radiol (2024) van der Graaf JW, van Hooff ML, van Ginneken B et al.

Supplementary material B:

Distribution of average manually measured Cobb angles.

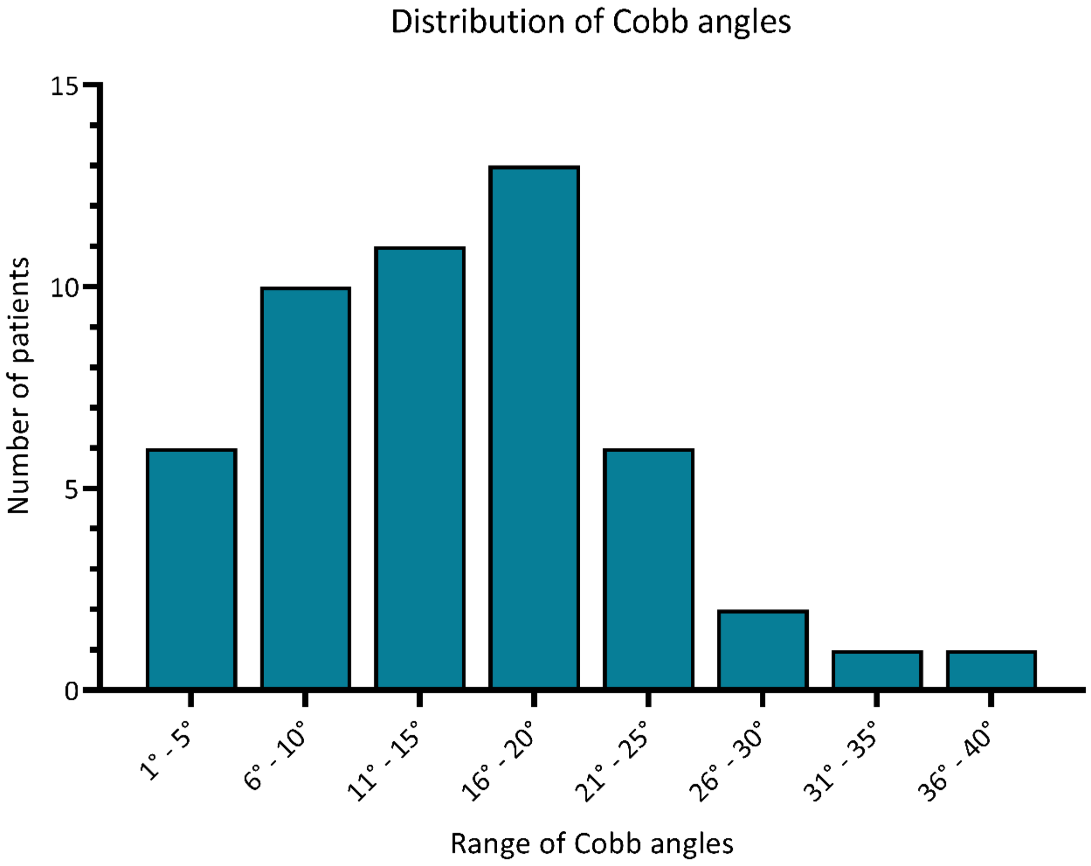

Supplementary material C:

All eight measurements were the difference between the reader (yellow) and the algorithm (red), when measured at the same vertebral levels as the reader, is larger than 5°.

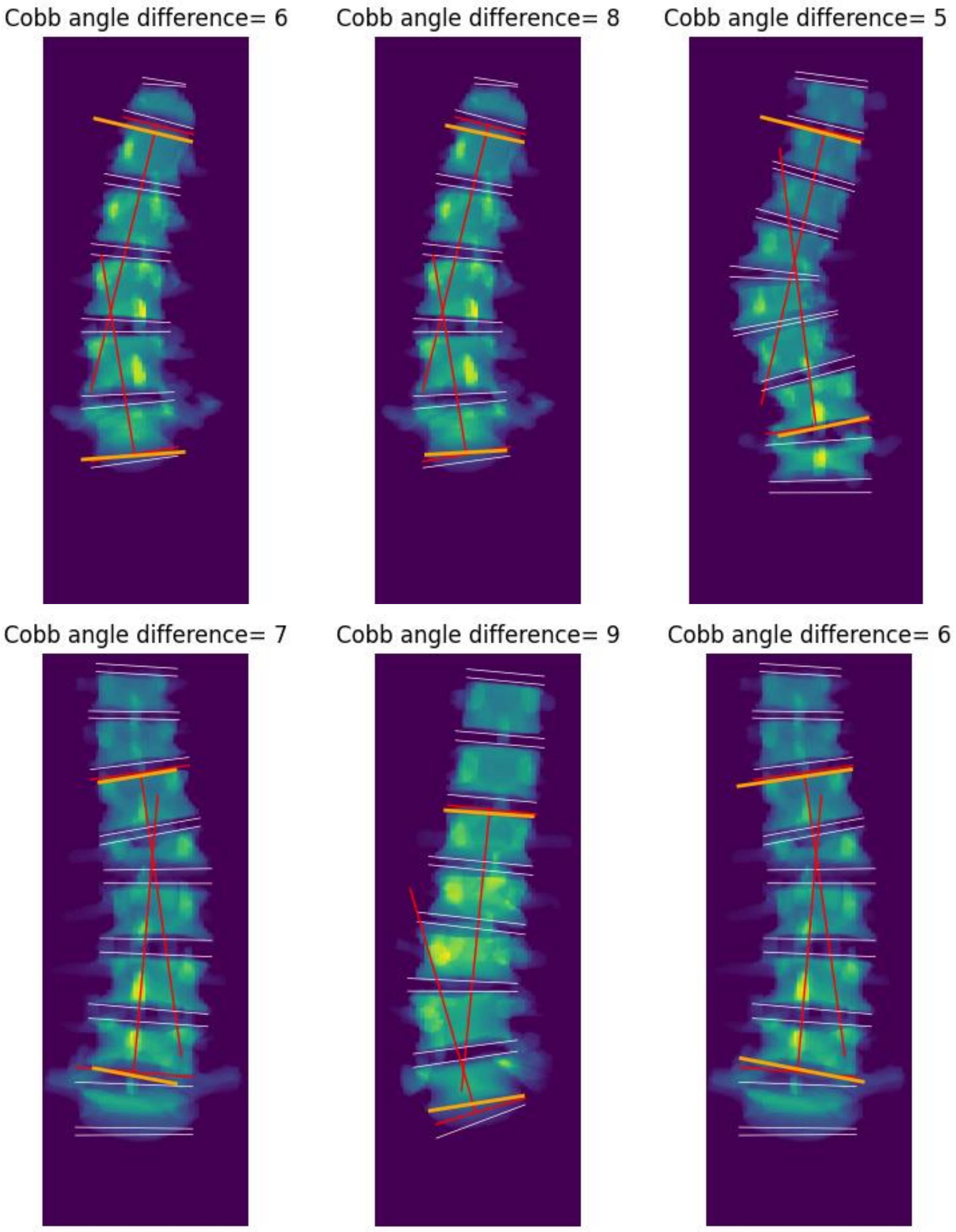

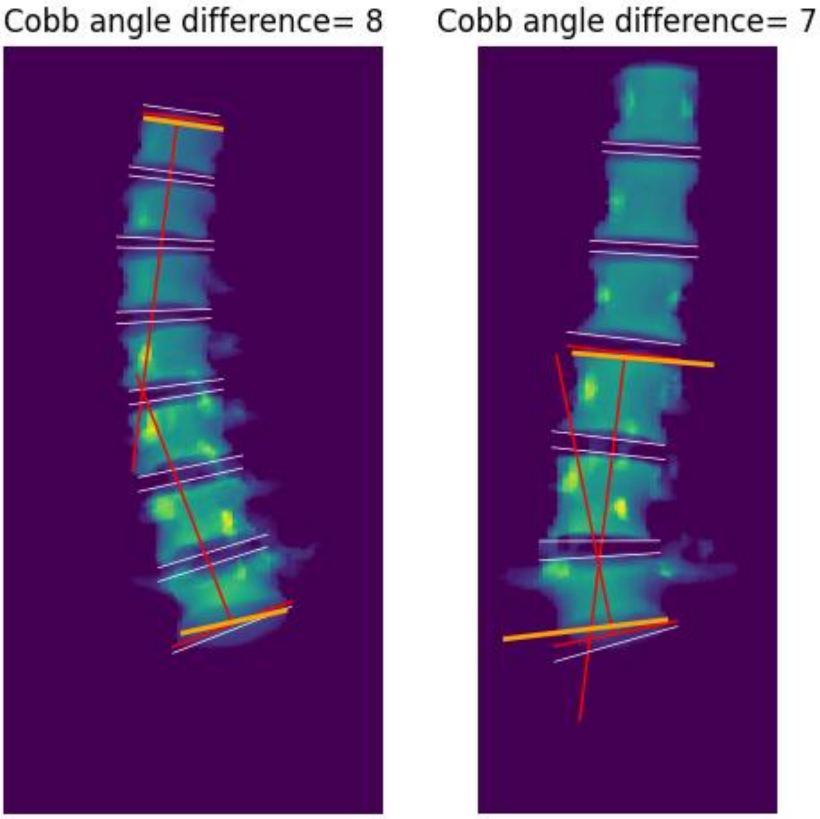

Eur Radiol (2024) van der Graaf JW, van Hooff ML, van Ginneken B et al.
